# Involvement of Brain-Enriched Guanylate Kinase-Associated Protein (BEGAIN) in Chronic Pain after Peripheral Nerve Injury

**DOI:** 10.1523/ENEURO.0110-16.2016

**Published:** 2016-10-17

**Authors:** Tayo Katano, Masafumi Fukuda, Hidemasa Furue, Maya Yamazaki, Manabu Abe, Masahiko Watanabe, Kazuhiko Nishida, Ikuko Yao, Akihiro Yamada, Yutaka Hata, Nobuaki Okumura, Takanobu Nakazawa, Tadashi Yamamoto, Kenji Sakimura, Toshifumi Takao, Seiji Ito

**Affiliations:** 1 Department of Medical Chemistry, Kansai Medical University, Hirakata 573-1010, Japan; 2 Laboratory of Protein Profiling and Functional Proteomics, Institute for Protein Research, Osaka University, Suita 565-0871, Japan; 3 Division of Neural Signaling, Department of Information Physiology, National Institute for Physiological Sciences, Okazaki 444-8787, Japan; 4 Department of Cellular Neurobiology, Brain Research Institute, Niigata University, Niigata 951-8585, Japan; 5 Department of Neurology, University of California, San Francisco, CA 94158; 6 Department of Anatomy, Hokkaido University School of Medicine, Sapporo 060-8638, Japan; 7 Department of Optical Imaging, Institute for Medical Photonics Research, Preeminent Medical Photonics Education & Research Center, Hamamatsu University School of Medicine, Hamamatsu, 431-3192, Japan; 8 Department of Medical Biochemistry, Graduate School of Medicine, Tokyo Medical and Dental University, Tokyo 113-8519, Japan; 9 Laboratory of Homeostatic Integration, Institute for Protein Research, Osaka University, Suita 565-0871, Japan; 10 Drug Innovation Center, Graduate School of Pharmaceutical Science, Osaka University, Suita, 565-0871, Japan; 11 Cell Signal Unit, Okinawa Institute of Science and Technology Graduate University, Okinawa 904-0495, Japan

**Keywords:** BEGAIN, GluN2B, neuropathic pain, proteomics, postsynaptic density, spinal lamina II

## Abstract

Maintenance of neuropathic pain caused by peripheral nerve injury crucially depends on the phosphorylation of GluN2B, a subunit of the N-methyl-d-aspartate (NMDA) receptor, at Tyr1472 (Y1472) and subsequent formation of a postsynaptic density (PSD) complex of superficial spinal dorsal horn neurons. Here we took advantage of comparative proteomic analysis based on isobaric stable isotope tags (iTRAQ) between wild-type and knock-in mice with a mutation of Y1472 to Phe of GluN2B (Y1472F-KI) to search for PSD proteins in the spinal dorsal horn that mediate the signaling downstream of phosphorylated Y1472 GluN2B. Among several candidate proteins, we focused on brain-enriched guanylate kinase-associated protein (BEGAIN), which was specifically up-regulated in wild-type mice after spared nerve injury (SNI). Immunohistochemical analysis using the generated antibody demonstrated that BEGAIN was highly localized at the synapse of inner lamina II in the spinal dorsal horn and that its expression was up-regulated after SNI in wild-type, but not in Y1472F-KI, mice. In addition, alteration of the kinetics of evoked excitatory postsynaptic currents for NMDA but not those for α-amino-3-hydroxy-5-methyl-4-isoxazolepropionic acid (AMPA) receptors in spinal lamina II was demonstrated by BEGAIN deletion. We demonstrated that mechanical allodynia, a condition of abnormal pain induced by innocuous stimuli, in the SNI model was significantly attenuated in BEGAIN-deficient mice. However, there was no significant difference between naive wild-type and BEGAIN-knockout mice in terms of physiological threshold for mechanical stimuli. These results suggest that BEGAIN was involved in pathological pain transmission through NMDA receptor activation by the phosphorylation of GluN2B at Y1472 in spinal inner lamina II.

## Visual Overview

**Figure:**
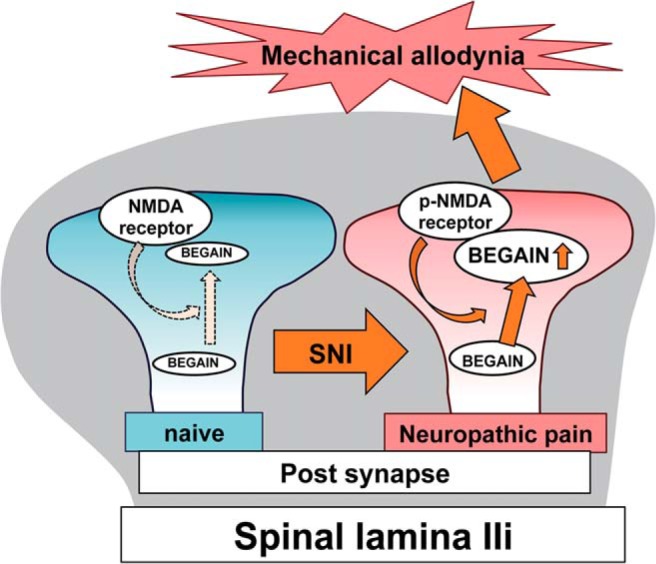

## Significance Statement

We for the first time reveal that brain-enriched guanylate kinase-associated protein (BEGAIN) plays a crucial role in pathological but not physiological pain. We previously demonstrated that neuropathic pain was attenuated in knock-in mice with Y1472F of GluN2B (Y1472F-KI). Here, by proteomic analysis of spinal dorsal horn, we found that the expression of BEGAIN protein was increased in wild-type, but not in Y1472F-KI, mice after peripheral nerve injury. BEGAIN was localized at synapses in lamina IIi of the spinal dorsal horn. Moreover, neuropathic pain was significantly attenuated in the knockout mice of BEGAIN after peripheral nerve injury, demonstrating that BEGAIN was involved in pathological pain transmission through N-methyl-d-aspartate receptor activation after the phosphorylation of GluN2B at Y1472 in spinal lamina II.

## Introduction

Neuropathic pain is assumed to result from pathological neural plasticity caused by peripheral nerve injury. The pathological condition is accompanied by long-lasting abnormal pain, such as hyperalgesia or allodynia, which is maintained by multiple postsynaptic density (PSD) proteins in several areas of the brain and in spinal dorsal horn neurons. The superficial dorsal horn, for example laminae I–II, predominantly receives nociceptive inputs via primary afferent Aδ and C fibers; whereas low-threshold information targets deeper laminae (Todd, 2010; Braz et al., 2014). In the case of neuropathic pain, however, innocuous stimuli-triggered nociceptive pain is mediated by an abnormal pain circuit of the spinal dorsal horn, which is engaged in alteration of synaptic efficacy of interneurons in laminae IIi–IV, such as disruption of inhibitory control or facilitation of excitatory control (Braz et al., 2014; Duan et al., 2014; Peirs et al., 2015).

PSD proteins including N-methyl-d-aspartate (NMDA) receptors (NMDARs) and scaffold proteins participate in not only physiological pain but also abnormal pain transmission through the activation of intracellular signaling cascades in the spinal dorsal horn and brain (Craven and Bredt, 1998; Garner et al., 2000; Luo et al., 2014). The difference between physiological and pathological conditions of sensory transmission is determined by reversible change of the composition of PSD complexes in the spinal dorsal horn (Katano et al., 2008). The interaction between PSD-95 and GluN2B is accelerated after peripheral nerve injury (Peng et al., 2013). Also, a disruption of the PSD complex, such as the interaction between PSD-95 and GluN2B, attenuates neuropathic and inflammatory pain (Tao et al., 2001, 2003; D’Mello et al., 2011). These lines of evidence indicate that clarification of the alteration of proteins in the PSD complex is necessary for the elucidation of abnormal pain mechanisms as well as for drug discovery. However, with respect to neuropathic pain, such alteration in the spinal dorsal horn has so far not been fully reported.

Phosphorylation of GluN2B at Tyr1472 (Y1472) is crucial for maintenance of neuropathic pain in the spinal dorsal horn. The phosphorylation of GluN2B is increased in the spinal dorsal horn after peripheral nerve injury, which affects the localization of NMDARs containing GluN2B at the synapse and calcium influx via the receptor. On the other hand, inhibition of GluN2B phosphorylation in Fyn-knockout mice or in knock-in mice with the Y1472 site of GluN2B mutated to phenylalanine (Y1472F-KI) affects the localization of GluN2B at the center of the postsynapse and that of several calcium signaling proteins, thereby attenuating mechanical allodynia (Abe et al., 2005; Nakazawa et al., 2006; Matsumura et al., 2010; Katano et al., 2011). Furthermore, neural plasticity, long-term potentiation, and fear-related learning are impaired in Y1472F-KI mice (Nakazawa et al., 2006), suggesting that the phosphorylation of GluN2B at this site is indispensable for the neural plasticity involved in maintenance of neuropathic pain and formation of learning and memory. However, it remains unclear how PSD proteins maintain allodynia after the phosphorylation of GluN2B in the spinal dorsal horn, because many of the roles and functions of these proteins have not yet been identified.

Here, to identify novel proteins involved in the signaling cascades downstream of GluN2B phosphorylation at Y1472 with respect to neuropathic pain, we took advantage of proteomic screening of wild-type (WT) and Y1472F-KI mice before and after spared nerve injury (SNI). From these analyses, we identified brain-enriched guanylate kinase-associated protein (BEGAIN) as a neuropathic pain-related protein, which was specifically expressed in the spinal lamina IIi. This study is the first report on the role of BEGAIN in abnormal pain sensations after peripheral nerve injury *in vivo*. Some of the data in this paper have been presented in abstract form at Society for Neuroscience meetings (Katano et al., 2012, 2015).

## Materials and Methods

### Animals and behavioral studies

GluN2B Y1472F-KI mice were produced by a gene-targeting technique as reported previously (Nakazawa et al., 2006). BEGAIN-floxed (BEGAIN^flox/+^) mice were produced by using the embryonic stem (ES) cell line RENKA, which was derived from the C57BL/6N strain (Mishina and Sakimura, 2007). Homologous recombinants among the ES cells were identified by Southern blot analysis. To yield heterozygous knockout (BEGAIN^+/–^) mice, BEGAIN^flox/+^ mice were crossed with TLCN-Cre mice, by which recombination is induced throughout the whole body (Nakamura et al., 2001; Mishina and Sakimura, 2007).

The neuropathic pain model of SNI was made according to the procedure reported previously (Decosterd and Woolf, 2000) with a slight modification (Katano et al., 2011). In the behavioral study, mice were randomly placed individually in a plastic case, which was placed on a mesh floor or plantar plate. Before each test, the mice were habituated for 0.5–1 h to allow acclimatization to the test environment. Mechanical threshold or allodynia elicited by SNI was assessed by use of the von Frey test. Each test was started from an initial filament (0.008 g). The filaments were inserted through the mesh floor of the cage and applied in ascending order five times at an interval of a few seconds to the plantar surface of the hindpaw ipsilateral to the operation side. The threshold was taken as the lowest force required for a withdrawal reflex of the paw to one of five repetitive stimuli, with the cutoff set at 2 g (Tal and Bennett, 1994; Decosterd and Woolf, 2000). Following Chaplan’s up-down methods, the threshold was further confirmed by additional tests showing the positive and negative responses by using upper and lower filaments, respectively (Chaplan et al., 1994).

### Antibodies

Rabbit anti-BEGAIN C17 and goat anti-PKCγ C14 antibodies were raised against C-terminal peptide SRKDSLTKAQLYGTLLN of mouse BEGAIN and mouse PKCγ (Yoshida et al., 2006), respectively. Commercially available antibodies against PSD-95 (Upstate Biotechnology, Lake Placid, NY), Hsp60 (Upstate Biotechnology), GluN2B (Millipore, Bedford, MA), synaptophysin (Millipore), glial fibrillary acidic protein (GFAP; Millipore), β-tubulin (Sigma, St. Louis, MO), and isothiocyanate-conjugated *Bandeiraea simplicifolia* isolectin B4 (IB4, Sigma) were also used.

### Drug administration

Intrathecal (i.t.) injection was performed as described previously (Minami et al., 1995). A 27-gauge stainless-steel needle (0.35 mm outer diameter) attached to a microsyringe was inserted between the L5 and L6 vertebrae of conscious mice, and Ro25-6981 (100 μg/mouse, Tocris Bioscience, Bristol, UK; Mihara et al., 2011; Kim et al., 2012) in 1% dimethylsulfoxide/saline (5 μl) was injected i.t. 0.5 h before assessment of mechanical allodynia. Attenuation of the withdrawal threshold for mechanical allodynia by Ro25-6981, which is a specific antagonist of NMDAR containing GluN2B, was measured three times 30–90 min after the injection.

### Subcellular fractionation of spinal dorsal horn for Western blotting

After anesthesia with isoflurane, the spinal dorsal horn at L4–L6 levels was collected and homogenized with a Potter-Elvehjem homogenizer in 20 mm Tris-HCl (pH 8.0) containing 0.32 m sucrose, 2 mm dithiothreitol, protease inhibitor cocktail (Sigma), and phosphatase inhibitor (Nacalai Tesque, Kyoto, Japan). After centrifugation of the homogenate at 800 × *g* for 10 min, the pellet (P1; nuclear) and supernatant (S1) were separated. The S1 fraction was then centrifuged at 13,800 × *g* for 20 min. The precipitate (P2), the membrane fraction, was suspended in 20 mm Tris-HCl (pH 8.0), after which an equal volume of 1% Triton X-100 in 20 mm Tris-HCl (pH 8.0) was added to it. The membrane fraction was rotated for 15 min at 4°C and then centrifuged for 20 min at 15,000 rpm. The resulting pellet was used as the crude PSD fraction (cPSD).

### Preparation of PSD fractions from spinal dorsal horn for proteomic analysis

After anesthesia with isoflurane, more than 100 male 8- to 10-week-old mice from four groups comprising WT naive, WT SNI at day 7, Y1472F-KI naive, and Y1472F-KI SNI at day 7 were killed, and their lumbar spinal dorsal horns at L4–L6 were collected. The PSD fraction was prepared essentially as described by Carlin et al. (1980) with slight modifications (Carlin et al., 1980). For preparation of PSD fractions, lumbar spinal dorsal horns were homogenized in solution A [0.32 m sucrose, 1 mm NaHCO_3_, 1 mm MgCl_2_, 0.5 mm CaCl_2_, 1 mm Na_3_VO_4_, and protease inhibitor cocktail (Sigma)] with a Potter-Elvehjem homogenizer. The pellet obtained by centrifugation of the S1 fraction at 13,800 × *g* for 20 min was used as the P2 fraction. This fraction was then suspended in solution B (0.32 m sucrose containing 1 mm NaHCO_3_) and applied onto a discontinuous sucrose gradients composed of 3.4 ml of 1.2 m, 3.4 ml of 1.0 m, and 3 ml of 0.85 m sucrose in 1 mm NaHCO_3_ in tubes. The tube was centrifuged at 82,500 × *g* for 120 min. The interface between 1.0 and 1.2 m sucrose was collected and dissolved for 15 min with buffer C consisting of 0.5% Triton X-100 and 6 mm Tris-HCl (pH 8.0). For collection of the insoluble fraction, the interface fraction was centrifuged at 32,800 × *g* for 30 min. The insoluble fraction was dissolved in 7 m urea, 2 m thiourea, 4% CHAPS, and 2% SDS and used as the PSD fraction. The purity of the PSD fraction was confirmed by Western blotting with anti–PSD-95 and anti-Hsp60 antibodies.

### Western blot analysis

In each figure, all subcellular fractions or homogenates from different tissues were subjected to a single gel for SDS-PAGE (10 or 12.5% acrylamide), and the separated proteins were transferred to a polyvinylidene fluoride membrane. After blocking for 1 h at room temperature with 3% skim milk or 3% bovine serum albumin in TBS-T buffer consisting of 0.1% Triton X-100, 150 mm NaCl, and 10 mm Tris-HCl (pH 7.5), the membrane was incubated at 4°C overnight with rabbit anti-BEGAIN (0.5 μg/ml), anti–PSD-95 (1:1000), anti-Hsp60 (1:1000), or anti–β-tubulin (1:2000) antibodies. The membrane was washed with the TBS-T buffer and incubated for 1 h with horseradish peroxidase–conjugated goat anti-rabbit IgG (1:20,000; Zymed, San Francisco, CA) or goat anti-mouse IgG (1:20,000; GE Healthcare, Chicago, IL). It was then washed four times with TBS-T buffer. Immunoreactivity was detected by use of an enhanced chemiluminescence detection kit (Chemi-Lumi One Super; Nacalai Tesque) after incubation with horseradish peroxidase–conjugated goat anti-rabbit IgG (1:20,000; Zymed) or goat anti-mouse IgG (1:20,000; GE Healthcare). Detection of several proteins, such as BEGAIN, PSD-95, Hsp60, GluN2B, and β-tubulin, in a single gel was performed sequentially. That is, the preceding antibody was stripped from the polyvinylidene fluoride membrane, which was then reprobed with another primary antibody for the detection of the next protein.

### In-gel digestion and iTRAQ labeling

PSD fractions (25 μg) prepared from the four groups were separated on 10% SDS-PAGE gels without stacking gel; after Coomassie Brilliant Blue staining, the lanes were separated into six parts according to molecular-weight range: <50, 50 to 74, 75 to 99, 100 to 149, 150 to 249, and >250 kDa. In-gel digestion was performed for the PSD proteins in these 24 gel parts in individual tubes by using 10 mm dithiothreitol for reduction, 100 mm acrylamide for alkylation, 500 nm trypsin for digestion, and 500 mm triethylammonium bicarbonate as the buffer for all the reactions. Tryptic peptides were extracted from the gel pieces with 50% acetonitrile (ACN) in 0.1% trifluoroacetic acid (TFA) and lyophilized. Digested samples were labeled with the isobaric stable isotope tags (114-, 115-, 116-, and 117-iTRAQ reagents) for comparative quantitation according to the manufacturer’s instruction (Applied Biosystems, Foster City, CA). The four iTRAQ-labeled samples with the same molecular weight range were combined after having been diluted 10 times with 10 mm KH_2_PO_4_ (pH 3.0) in 25% ACN. The combined sample was purified by cation-exchange chromatography (cartridge column kit; Applied Biosystems), followed by desalting with a Bond Elut C18 cartridge column (Agilent Technologies, Santa Clara, CA). The eluate was dried by vacuum centrifugation, dissolved in 0.1% TFA in 50% ACN, and diluted 10 times with 0.1% TFA for further analysis.

### Liquid chromatography/matrix-assisted laser desorption ionization mass spectrometry and tandem mass spectrometry analysis

The combined samples were applied to a Prominence nano-liquid chromatography System (Shimadzu, Kyoto, Japan) coupled to an AccuSpot LC spotting system (Shimadzu). The mobile phases were solvent A [0.1% TFA in water/ACN (95:5, v/v)] and B [0.1% TFA in water/ACN (10:90, v/v)]. Peptide digests were adsorbed and desalted on a precolumn (Monolith, 0.2 × 100 mm; Kyoto Monotech, Kyoto, Japan) with 0.1% TFA in water at a flow rate of 35 μl/min (0–5 min). Peptide digests were then separated on an analytical column (Monolith, 0.1 × 250 mm) at a flow rate of 1 μl/min with a gradient obtained by changing the ratio of solvent B (%) as follows: 5 min, 10% (initial %); 7 min, 10%; 10 min, 15%, 36 min, 40%; 40 min, 60%; 41 min, 95%; 59 min, 95%; 60 min, 10%; and 77 min, 10%. The eluate was recorded at 210 nm, mixed with matrix-assisted laser desorption ionization (MALDI) matrix solution [5 mg/ml *α*-cyano-4-hydroxycinnamic acid in 60% (v/v) ACN containing 0.1% TFA], and directly spotted onto a 192-well MALDI plate (Applied Biosystems). Mass spectrometry and tandem mass spectrometry (MS/MS) analyses were performed on a 4700 Proteomics Analyzer (Applied Biosystems).

### Identification and quantitative analysis

All MS/MS spectra were combined, processed, database-searched, and subjected to comparative quantification with ProteinPilot software (version 2.0.1; Applied Biosystems), in which the Paragon algorithm is used to carry out database matching for protein identification based on a novel small sequence tag search method with simple search criteria (Shilov et al., 2007). For identification, “Gel-based ID” was selected in the software, with propionamide for Cys alkylation. The UniProt protein database, which had been downloaded in March 2009 from a website and updated on a regular basis, was used for database searching. To demonstrate search results, we adopted the identified that showed more than 95% confidence identification (Paragon algorithm) based on their MS/MS spectra and had a low false discovery rate (<1.0%). For scoring, the MS/MS spectra that have not already been used to justify an already assigned more confident protein only contributed to the “Unused ProtScore.” Therefore, identified proteins were reported only if they had a sufficient Unused ProtScore as top hit removing redundant protein entries. Bias correction was performed by using ProteinPilot software as normalization for comparative quantification to correct the median ratio to unity, based on the assumption that most proteins do not change in expression. Namely, the observed iTRAQ ratios of each peptide were divided by the corresponding median values of the iTRAQ ratios so that unequal mixing of the different labeled samples could be corrected. Identified proteins with iTRAQ ratios below the low range (0.8) and *p*-value of <0.05 were considered to be underexpressed, whereas those above the high range (1.2) and *p*-value of <0.05 were considered to be overexpressed compared with those of the naive group. For evaluation of the expression level, some proteins extracted from two or more different gel parts of molecular range were excluded. The mass spectrometry proteomics data were deposited to the Peptide Atlas (http://www.peptideatlas.org) with the dataset identifier PASS00929.

### Histochemistry

Animals were anesthetized by an intraperitoneal administration of sodium pentobarbital (50 mg/kg) and perfused with 4% paraformaldehyde in 0.12 m sodium phosphate (pH 7.4). After dissection, spinal cords were postfixed for 4 h in the same fixative at 4°C and cryoprotected overnight in 30% (w/v) sucrose in PBS (–) at pH 7.4. Transverse sections (14 μm thick) of the spinal cord at L4–L6 were cut on a cryostat and processed for immunohistochemistry with anti-BEGAIN (1 μg/ml), anti-PKCγ (1 μg/ml), anti–PSD-95 (1 μg/ml), anti-synaptophysin (1:1000), or anti-GFAP (1:500) as primary antibody overnight at 4°C after antigen retrieval at 110°C for 15 min in citrate buffer (pH 6.0). Thereafter, the sections were incubated with Alexa Fluor 488–, 546–, or 633–conjugated goat anti-mouse, -rabbit, or -goat IgG as secondary antibody (1:300–500, Invitrogen) for 90 min at room temperature. For the fluorescein IB4 staining condition, sections of spinal cords were preincubated for 20 min at room temperature with 0.1% Triton X-100 in PBS supplemented with 0.1 mm CaCl_2_, 0.1 mm MgCl_2_, and 0.1 mm MnCl_2_ after antigen retrieval. Then, the sections were incubated with 50 μg/ml IB4 and anti-BEGAIN overnight at 4°C in above preincubation buffer containing 2% bovine serum albumin. Fluorescence images were captured with a Zeiss laser scanning confocal microscope (LSM700), and quantification of fluorescent signals was carried out by use of ImageJ software. The colocalization analysis was performed by use of Imaris (Bitplane, South Windsor, CT; Costes et al., 2004; Marvizón et al., 2007). The *z*-series fluorescence images were captured, and three-dimensional data of regions of interest were automatically analyzed after threshold setting. The threshold was defined using two-dimensional histogram and Preview window in Imaris Coloc, which helps distinguish between low-intensity colocalization pixels of synaptic proteins and background (Costes et al., 2004). In the two-dimensional histogram, we chose pixels closest to the diagonal line. The statistical validity of colocalization was quantified by computing the Manders overlap coefficient.

### Reverse-transcription PCR for BEGAIN

The expression level of BEGAIN was determined in the spinal dorsal horn, dorsal root ganglion (DRG), and brain by performing reverse-transcription (RT)-PCR analysis. Total RNA was extracted by use of Trizol according to the manufacturer’s protocol (Invitrogen, San Diego, CA). First-strand cDNA for each tissue as a template was synthesized with Revatra Ace and oligo dT_20_. BEGAIN was detected by RT-PCR using primers (forward, ATTGACAAGCTGTCGGAGGA; and reverse, GGCAGCTCGGACACCTTAT).

### Electrophysiology

The electrophysiological recordings used for the current experiments were similar to those in an earlier study (Uta et al., 2010). Briefly, a 500-μm-thick transverse slice of mouse spinal cord was prepared 7 days after SNI and set in a chamber perfused with Krebs solution (in mm: NaCl 117, KCl 3.6, CaCl_2_ 2.5, MgCl_2_ 1.2, NaH_2_PO_4_ 1.2, NaHCO_3_ 25, and glucose 11) equilibrated with 95% O_2_-5% CO_2_ at 36°C. Patch-pipettes were filled with a solution having the following composition (in mm): Cs_2_SO_4_ 110, tetraethylammonium 5, CaCl_2_ 0.5, MgCl_2_ 2, EGTA 5, Mg-ATP 5, and HEPES 5 (pH 7.2 adjusted with CsOH). The pipettes had a resistance of 8–12 MΩ. Blind whole-cell voltage-clamp recordings were obtained from substantia gelatinosa (SG, lamina II) neurons. A monopolar stimulating electrode was put on the surface of the slice near the recording neuron to elicit evoked excitatory postsynaptic currents (EPSCs). Drugs were dissolved in Krebs solution and applied by bath-application.

### Statistical analysis

Data for immunohistochemistry and mechanical allodynia were expressed as the mean ± SEM and analyzed by using the unpaired *t* test, Mann–Whitney *U* test, or repeated-measures ANOVA followed by the Bonferroni *post hoc* test. *p* < 0.05 was considered to be statistically significant. Colocalization of synaptic proteins was quantified by Manders overlap coefficient analysis. Electrophysiological data were expressed as the mean ± SEM. Statistical significance was determined as *p* < 0.05 by using the paired *t* test.

## Results

### Attenuation of mechanical allodynia by lumbar intrathecal injection of Ro 25-6981, an antagonist of GluN2B-NMDAR, and in Y1472F-KI mice after SNI

**Figure 1.**
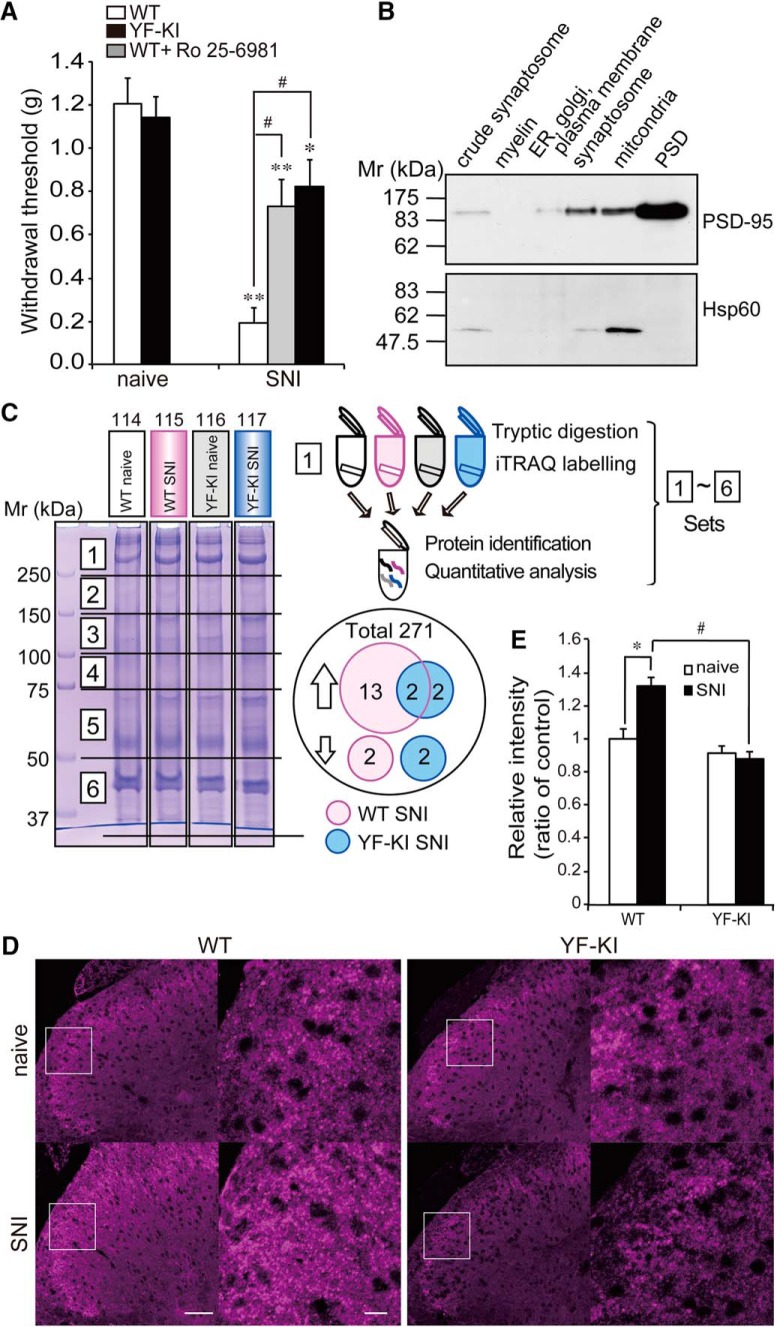
Behavioral analysis and identification of BEGAIN as a neuropathic pain–related protein in the postsynaptic density (PSD) fraction. ***A***, Attenuation of mechanical allodynia by inhibition of NMDAR containing GluN2B. Withdrawal thresholds of WT and Y1472F-KI (YF-KI) mice were assessed before and 7 days after SNI by use of von Frey filaments. The effect of the GluN2B antagonist Ro 25-6981 (100 μg/mouse) 7 days after SNI was assessed, and measurements were repeated three times within 30 and 90 min after i.t. injection of Ro 25-6981 at an interval of at least 15 min. The average was used as the value of the withdrawal threshold. Data are expressed as the mean ± SEM, *n* = 12. Significant differences are indicated: Mann–Whitney *U* test, *p < 0.05, **p < 0.01 vs. naive mice; ^#^*p* < 0.01 vs. WT SNI mice. ***B***, Purity of PSD fraction. PSD fractions were purified by sucrose-density gradient centrifugation and analyzed by Western blotting with anti–PSD-95 and anti-Hsp60 antibodies. PSD-95 is a marker protein of the PSD (upper panel), and Hsp60, for mitochondria (lower panel), as described in Materials and Methods. ***C***, SDS-PAGE of PSD fractions and workflow for quantitative proteomics using iTRAQ reagents. PSD fractions of four comparison groups were individually separated into six parts by 10% SDS-PAGE after Coomassie Brilliant Blue (CBB) staining. Proteins in each gel piece were digested and labeled with 4-plex iTRAQ reagents (114: WT naive/white, 115: WT SNI/magenta, 116: YF-KI naive/gray, and 117: YF-KI SNI/blue). Differentially labeled peptide samples in the same part were combined and subjected to subsequent protein identification and quantitative analysis. As a result of examination of all six parts, 271 proteins were identified and quantified. ***D***, Immunohistochemistry of BEGAIN in the spinal dorsal horn before and after SNI. Transverse sections (14 μm) of lumbar spinal cords prepared from WT and Y1472F-KI (YF-KI) mice before (naive) and 7 days after SNI were immunostained with anti-BEGAIN antibody. Higher magnification of the white boxes in the left images is shown as the right images. Scale bars, 50 and 10 μm. ***E***, Quantification of immunoreactivity of BEGAIN. Immunostaining intensity of WT and YF-KI was measured by using ImageJ. The intensity of immunofluorescence for the WT naive mice was taken as 1, and the data are expressed as the mean ± SEM (*n* = 23–31 slices from three mice for each group). Significant differences were determined by Mann–Whitney *U* test: *p < 0.05 vs. WT naive mice, ^#^*p* < 0.05 vs. WT SNI mice.

We previously demonstrated that phosphorylation of GluN2B at Y1472 was increased in the spinal dorsal horn of WT mice after spinal nerve transection and SNI (Matsumura et al., 2010; Katano et al., 2011) and that mechanical allodynia after the operations was significantly attenuated in Y1472F-KI mice. To clarify the participation of NMDAR activity of the spinal dorsal horn in neuropathic pain after the phosphorylation of its GluN2B subunit at Y1472, we prepared SNI-operated WT mice with or without i.t. injection of the GluN2B-specific antagonist Ro25-6981 into their lumbar spinal cord and SNI-operated Y1472F-KI mice, and analyzed their responses to mechanical stimulation (Fig. 1*A*). Seven days after SNI, the paw withdrawal threshold for the WT mice drastically dropped (0.19 ± 0.07 g, *n* = 12) compared with that for the naive mice (1.21 ± 0.12 g, *n* = 12). Ro25-6981 significantly attenuated the decrease in the threshold within 30–90 min after i.t. injection into the WT mice (0.73 ± 0.12 g, *n* = 12). Furthermore, the decrease in the withdrawal threshold for Y1472F-KI mice after SNI (0.82 ± 0.12 g, *n* = 12) was also partially attenuated compared with that for the WT mice after SNI. These results confirmed that the phosphorylation of GluN2B at Y1472 in the spinal dorsal horn was important for the maintenance of neuropathic pain even 7 days after SNI. Therefore we chose the spinal dorsal horn as the target tissue for our proteomic analysis.

**Figure 1-1.** Statistical table for behavioral analysis and immunohistochemistry. ***A***, Withdrawal threshold after SNI was significantly decreased in both WT and Y1472F-KI mice. In YF-KI and WT with Ro25-6981 after SNI, mechanical allodynia was significantly attenuated. ***E***, Fluorescence intensity of BEGAIN was specifically increased in the spinal dorsal horn in WT after SNI. Significant difference is indicated in ***A*** and ***E***.

Figure	Comparison	Mann–Whitney *U*	*n*
Fig. 1*A*, force (g)	WT naive vs. WT SNI	0.0002421	12 mice in one group
	WT naive vs. WT SNI + Ro25-6981	0.0102980	
	YF naive vs. YF SNI	0.0432658	
	WT SNI vs. WT SNI + Ro25-6981	0.0016202	
	WT SNI vs. YF SNI	0.0008131	
	YF SNI vs. WT SNI + Ro25-6981	0.3042951	
Fig. 1*E*, intensity of BEGAIN	WT naive vs. WT SNI	0.0487103	23–31 slices from three mice in each group
	WT SNI vs. YF-KI SNI	0.0487103	

**Figure 2.**
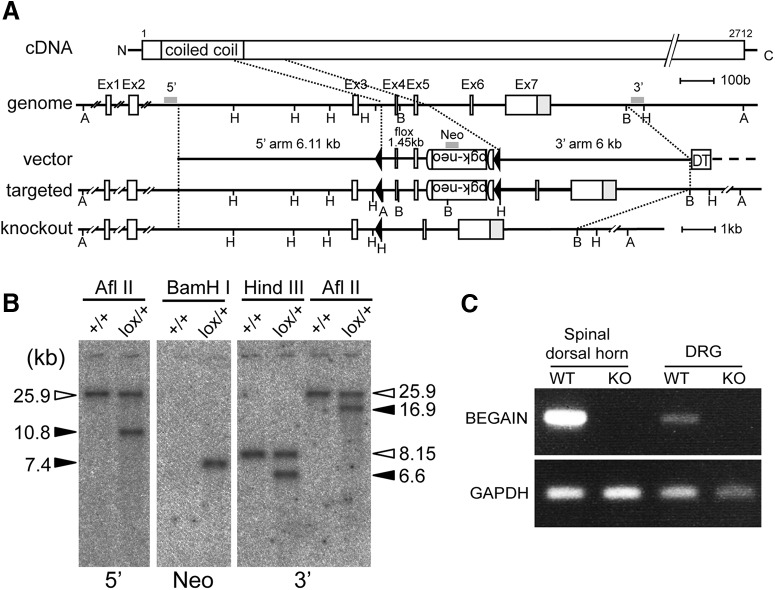
Generation of BEGAIN-KO mice. ***A***, Knockout strategy for the *begain* gene. Homologous recombination of the targeting plasmid resulted in insertion of the *pgk-neo* cassette (*neo*) and *loxP* sequences (filled triangles) into introns 3 and 5 of the *begain* gene. The floxed mice after germline transmission of ES cells with homologous recombination (Targeted) were crossed with Cre-deleter mice. Exons 4 and 5 of the *begain* gene were deleted from the germline (knockout) together with the *neo* cassette. A, AflII; B, BamHI; H, HindIII. ***B***, Southern blot analysis for homologous recombination of ES cells. Genomic DNA prepared from the WT (^+/+^) and begain^flox/+^ ES cells. Left, AflII-digested DNA hybridized with a 5′ probe: 25.9 kb for WT and 10.8 kb for floxed allele. Middle, BamHI-digested DNA hybridized with a Neo probe: 7.4 kb for floxed allele. Right, KpnI- or AflII-digested DNA hybridized with a 3′ probe: 25.9 or 8.15 kb, respectively, for WT and 16.9 or 6.6 kb, respectively, for floxed allele. ***C***, Expression of BEGAIN. RT-PCR of the spinal dorsal horn and DRG samples from WT and BEGAIN-KO (KO) mice.

**Figure 1-2.** Values of Auto Bias for iTRAQ analysis in ProteinPilot. Observed iTRAQ ratios of all peptides were normalized by using Auto Bias. Gel, a part of gel part in Figure 1*C*.

Gel	Auto bias (median)		
	115/114	116/114	117/114
1	1.3163	1.0467	1.0604
2	1.2254	1.0641	0.8925
3	0.9273	0.9753	1.0206
4	0.7433	1.0111	0.9129
5	0.998	0.9869	0.9197
6	0.9157	0.9237	0.9097

### Identification and quantitative analysis of PSD proteins in the spinal dorsal horn by proteomic analysis using isobaric stable isotope tags (iTRAQ)

To investigate the molecular mechanism of central sensitization via the phosphorylation of GluN2B in the spinal dorsal horn, we searched for novel proteins involved in neuropathic pain by comparative analysis using the proteomic approach. We chose the spinal dorsal horn as the target tissue for proteomic approach because mechanical allodynia caused by SNI was attenuated by intrathecal injection of Ro25-6981, a selective blocker of NMDAR containing GluN2B, into the lumbar spinal cord (Fig. 1*A*). Differential analysis of the lumbar spinal dorsal horn among four groups—WT naive, WT SNI, Y1472F-KI naive, and Y1472F-KI SNI—was performed by using the proteomic approach. We purified the PSD fraction of the lumbar spinal dorsal horn from more than 100 mice in each group by sucrose-density gradient. The purity was confirmed by Western blotting with anti–PSD-95 and Hsp60 antibodies, which labeled the PSD and mitochondrial fraction, respectively (Fig. 1*B*). Each PSD fraction was solubilized and separated by molecular size–dependent fractionation using SDS-PAGE (Fig. 1*C*, six pieces of gels from a given lane). The tryptic peptides derived from PSD proteins in six corresponding gel pieces from each group were labeled with 4-plex iTRAQ reagents (114, WT naive; 115, WT SNI; 116, Y1472F-KI naive; and 117, Y1472F-KI SNI). MS/MS spectra of the labeled peptides were processed and database-searched with ProteinPilot software, and protein amounts were quantified based on labeled iTRAQ peaks. In particular, we used peptides with more than 95% confidence and <1% false discovery rate for protein identification. By using these criteria, 208 and 63 proteins including receptors, channels, kinases, and scaffold proteins were identified from single and multiple gel pieces, respectively (Fig. 1*C* and PASS00929 in Peptide Atlas). For quantitative analysis, the median was used for normalization of the iTRAQ ratios as described in Materials and Methods. The level of two proteins (Ras/Rap GTPase-activating protein SynGAP; Protein piccolo) was increased in both WT and Y1472F-KI mice after SNI (Table 1 and PASS00929 in Peptide Atlas). These proteins were identified as neuropathic pain–related proteins independent of GluN2B phosphorylation at Y1472. Moreover, we specifically demonstrated increased expression of two proteins (IQ motif and SEC7 domain-containing protein 2, SH3; multiple ankyrin repeat domains protein 2) and decreased expression of two proteins (Actin cytoplasmic 2; Voltage-dependent anion-selective channel protein 1) in Y1472F-KI mice after SNI compared with their expression in naive mice, and thus that their levels were affected by the attenuation of GluN2B phosphorylation at Y1472 after SNI. Thirteen upregulated proteins, including CaMKII, and two downregulated proteins (pyruvate dehydrogenase E1 component subunit β; d-β-hydroxybutyrate dehydrogenase) were found only in the WT SNI group (Fig. 1*C*, Table 1, and PASS00929 in Peptide Atlas). Among these former proteins, one of them, i.e., BEGAIN, was reported to interact with PSD-95 in the brain; however, its molecular function in the brain and spinal cord have not yet been reported. Here, we focused on BEGAIN, whose expression was increased in WT mice (1.34-fold vs. WT naive; Table 1), but not in Y1472F-KI mice, after SNI.

**Table 1.** List of proteins whose expression was increased specifically in the WT or in both WT and Y1472F-KI SNI mice.

Accession no.	Name	Isotope reagent 115/114				Isotope reagent 117/116			
		Ratio	*p*	Lower CI	Upper CI	Ratio	*p*	Lower CI	Upper CI
Q68EF6	Brain-enriched guanylate kinase-associated protein	1.3*	0.045	1.011	1.798	1.1	0.258	0.880	1.463
P28652	Calcium/calmodulin-dependent protein kinase type II beta chain	1.2*	0.031	1.018	1.385	1.1	0.067	0.990	1.283
Q923T9	Calcium/calmodulin-dependent protein kinase type II gamma chain	1.2*	0.008	1.060	1.363	1.1	0.094	0.977	1.285
Q6P9K8	Caskin-1	1.6*	0.001	1.403	1.732	1.1	0.512	0.769	1.526
Q6PFD5	Disks large-associated protein 3	1.2*	0.039	1.009	1.344	1.1	0.124	0.974	1.211
P05064	Fructose-bisphosphate aldolase A	1.2*	0.011	1.046	1.317	1.1	0.364	0.920	1.230
P16858	Glyceraldehyde-3-phosphate dehydrogenase	1.3*	0.000	1.178	1.426	1.1	0.137	0.974	1.198
P20357	Microtubule-associated protein 2	1.194*	0.005	1.081	1.319	1.217	0.096	0.954	1.551
P24369	Peptidyl-prolyl cis-trans isomerase B	1.265*	0.036	1.067	1.500	0.912	0.806	0.022	38.176
P15331	Peripherin	1.210*	0.020	1.039	1.409	1.085	0.192	0.952	1.236
Q9QYX7	Protein piccolo	1.179*	0.010	1.046	1.328	1.173*	0.011	1.042	1.321
Q9EQZ6	Rap guanine nucleotide exchange factor 4	1.201*	0.003	1.087	1.326	1.042	0.464	0.922	1.177
F6SEU4	Ras GTPase-activating protein SynGAP	1.194*	0.016	1.037	1.373	1.225*	0.002	1.088	1.381
Q64332	Synapsin-2	1.4*	0.036	1.052	1.844	1.2	0.48	0.434	3.469
P42669	Transcriptional activator protein Pur-alpha	1.173*	0.001	1.090	1.262	0.873	0.028	0.777	0.981

The expression level of 13 proteins was specifically increased in the WT after SNI. Two proteins were increased in both WT and Y1472F-KI mice. Lower CI is the lower bound of the confidence interval for the average ratio; upper CI is the upper bound of the confidence interval for the average ratio. *Significant increase.

### Difference in protein expression of BEGAIN in the spinal dorsal horn between WT and Y1472F-KI mice after SNI

In the proteomic analysis, we identified BEGAIN, CaMKII, and others whose expression was specifically increased in the PSD fraction of the spinal dorsal horn of WT SNI mice. The amount and post-translational modification of CaMKII and other proteins in the superficial spinal dorsal horn are increased after nerve injury (Katano et al., 2008; Peng et al., 2013). Thus, to further confirm the relative abundance and localization of BEGAIN in the spinal cord, we determined its expression by immunohistochemistry using anti-BEGAIN antibody generated in this study. The fluorescence intensity of BEGAIN signals significantly increased in the superficial dorsal horn of WT mice after SNI (Fig. 1*D*,*E*, 1.32 ± 0.05, *n* = 23–38 slices from three mice, *p* < 0.05), whereas there was no significant difference between the intensity before and 7 days after SNI in the Y1472F-KI mice (Fig. 1*D*,*E*, naive: 0.92 ± 0.04 vs. SNI: 0.88 ± 0.04, *n* = 31 slices from three mice). These results suggest that protein expression of BEGAIN in the spinal dorsal horn was affected (i.e., increased) by the phosphorylation of GluN2B at Y1472 in the spinal dorsal horn after peripheral nerve injury.

### Generation of BEGAIN knockout mouse

To clarify the role of BEGAIN in pain transmission after peripheral nerve injury, we generated knockout mice. The targeting vector construct was designed as shown in Fig. 2*A*. Homologous recombination in ES cells was confirmed by Southern blot analysis (Fig. 2*B*). BEGAIN^flox/+^ mice were crossed with TLCN-Cre mice (Nakamura et al., 2001; Fuse et al., 2004), and BEGAIN ^–/+^ mice were further interbred to generate BEGAIN^–/–^ mice (BEGAIN-KO). BEGAIN-KO mice showed the normal Mendelian ratio of offspring (1:2.06:0.98 based on 657 mice; Table 2) after breeding of BEGAIN^–/+^ mice. BEGAIN deletion in the spinal dorsal horn and DRG was confirmed by RT-PCR using WT and BEGAIN-KO mice. BEGAIN mRNA was completely missing in the spinal dorsal horn and DRG in the BEGAIN-KO mice (Fig. 2*C*). The amount of BEGAIN mRNA in the DRG was much less than that in the spinal dorsal horn, suggesting that the BEGAIN protein in the spinal cord was mainly expressed in spinal cord neurons.

**Table 2.** Mendelian ratio of BEGAIN knockout mice.

Mouse	Number	Rate	Theoretical rate	Theoretical value	χ^2^ test
Total	657	—	—	—	—
WT	153	1	1	164.25	
Heterozygous	330	2.06	2	328.5	
KO	174	0.98	1	164.25	0.50759

BEGAIN-KO mice were born at the predicted Mendelian ratio by interbreeding of BEGAIN heterozygous knockout mice. The ratio was analyzed by use of the χ^2^ test.

### Expression and distribution of BEGAIN in the CNS

**Figure 3.**
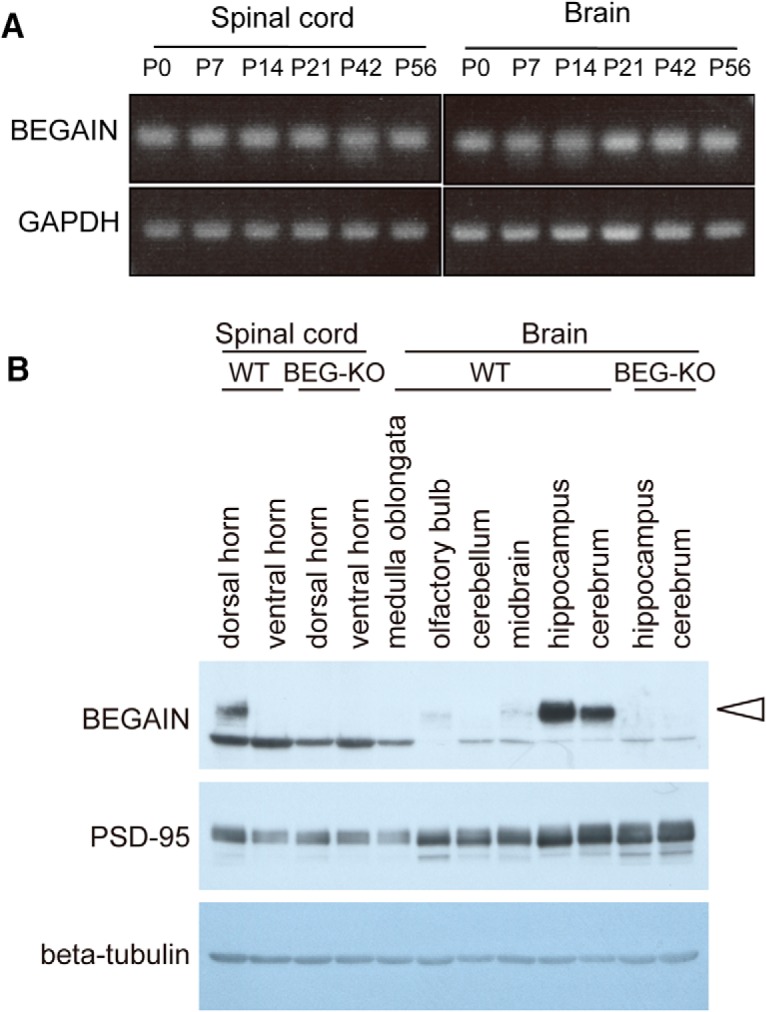
Expression of BEGAIN in the CNS. ***A***, Expression of BEGAIN during postnatal development. RT-PCR of the spinal cord and brain at postnatal developmental stages between day 0 and day 56. ***B***, Detection of BEGAIN in the CNS. Western blot analysis of the CNS (30 μg each) in WT and BEGAIN-KO (BEG-KO), performed by use of 10% SDS-PAGE. Results for BEGAIN (arrowhead), PSD-95, and β-tubulin are shown.

In a previous study, BEGAIN mRNA was detected in the whole brain, but not in other tissues, such as heart, spleen, lung, liver, kidney, skeletal muscles, and testis (Deguchi et al., 1998). However, its expression in the spinal cord had not been clarified. By RT-PCR, we analyzed the expression in the spinal cord and brain during postnatal development, because we detected it in the spinal dorsal horn for the first time in this report. The expression was maintained at a nearly equal level during the postnatal development stages from day 0 to day 56 (Fig. 3*A*). We next analyzed the distribution of BEGAIN protein in adult mice by performing Western blotting. Mouse BEGAIN comprises 600 amino acids (accession no. Q68EF6), and it was found to have a molecular mass of 65 kDa by Western blotting (Fig. 3*B*, arrowhead). Because the band was observed in the spinal dorsal horn but not in the ventral horn (Fig. 3), it seemed that BEGAIN was involved in sensory transmission but not in motor neuron functions. On the other hand, BEGAIN was strongly detected in the cortex and hippocampus, but not in the cerebellum and medulla oblongata (Fig. 3*B*, WT). Thus, in our Western blot analysis, BEGAIN was expressed in the spinal dorsal horn and restricted regions in the brain, and the expression was lost in the BEGAIN-KO mice (Fig. 3*B*).

### Concentration of BEGAIN at the synapse in spinal lamina II

**Figure 4.**
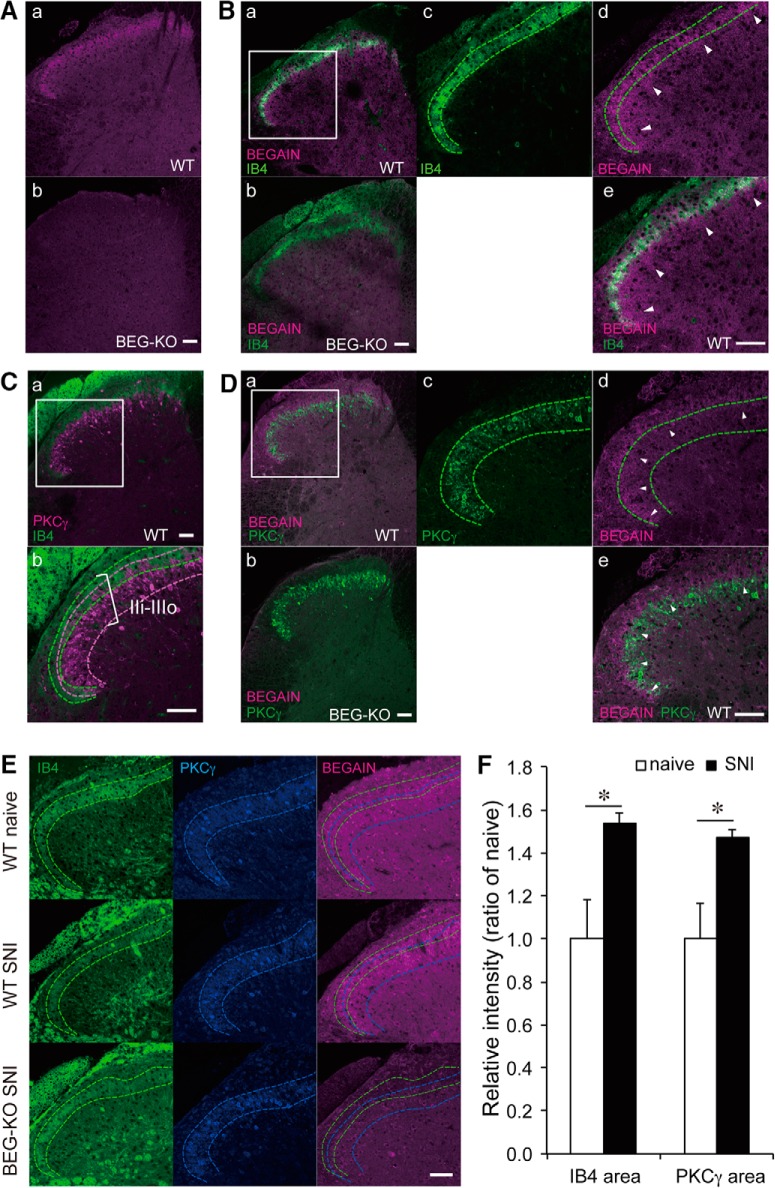
Localization of BEGAIN in the spinal dorsal horn. ***A***, Detection of BEGAIN in the spinal dorsal horn. Transverse sections (14 μm) of lumbar spinal cords prepared from WT (***a***) and BEGAIN-KO (BEG-KO; ***b***) mice were immunostained with anti-BEGAIN antibody. ***B***, Double-staining of the spinal dorsal horn of WT (***a***, ***c–e***) and BEG-KO (***b***) mice by using anti-BEGAIN antibody and IB4, a marker of nonpeptidergic afferents in lamina IIi. ***c–e***, High magnification of the white box in ***a***. Green lines indicate IB4-positive area. Arrowheads indicate BEGAIN single-positive area (***d*** and ***e***). ***C***, Lamina IIi–IIIo, labeled with IB4 and anti-PKCγ antibody in WT mice (***a*** and ***b***). Higher magnification of the white box in ***a*** is shown in ***b***. Green and magenta lines indicate IB4- and PKCγ-positive areas, respectively. ***D***, Double-staining of the spinal dorsal horn of WT (***a***, ***c–***e) and BEG-KO (***b***) mice by use of anti-BEGAIN and PKCγ antibodies. Green lines indicate PKCγ-positive area. Arrowheads indicate BEGAIN and PKCγ double-positive area (***d*** and ***e***). ***E***, Triple-staining of the spinal dorsal horn of WT naive, WT SNI, and BEG-KO SNI. Green and blue lines indicate IB4- and PKCγ-positive areas, respectively. Scale bars, 50 μm. ***F***, Quantification of immunostaining intensity of BEGAIN in IB4- and PKCγ-positive areas. The signal of BEGAIN in each area was measured before and after SNI by using ImageJ. The intensity of immunofluorescence for the naive mice was taken as 1, and the data are expressed as the mean ± SEM (*n* = 31–39 slices from three mice for each group). Significant differences, determined by use of Mann–Whitney *U* test, are indicated: *p < 0.05 vs. WT naive mice.

The spinal dorsal horn is organized into laminae I–VI, where peripheral inputs are received from different types of fibers: Aβ, Aδ, and C (Rexed, 1952). To clarify the participation of BEGAIN in abnormal pain transmission, we analyzed the localization of BEGAIN in these laminae by performing immunohistochemistry using anti-BEGAIN antibody. BEGAIN was detected in the superficial area of the spinal dorsal horn in WT mice but not in BEGAIN-KO mice (Fig. 4*A*). BEGAIN was colocalized with an IB4 lectin-binding nonpeptidergic population (Fig. 4*Ba*,*Be*), which targets the dorsal part of lamina IIi (Fig. 4*C*). PKCγ was concentrated in the ventral part of laminae IIi and IIIo (Fig. 4*C*; Polgár et al., 1999; Neumann et al., 2008). BEGAIN was highly concentrated in the IB4-positive area (Fig. 4*Bd*,*Be*); however, its signal was also detected in the PKCγ-positive area (Fig. 4*Dd*,*De*, arrowheads), where myelinated fibers terminate (Fig. 4*C*). These results indicate that BEGAIN-positive neurons may receive not only nociceptive but also innocuous stimuli from primary afferent fibers. On the other hand, the localization of IB4 and PKCγ in the spinal dorsal horn was not affected in BEGAIN-KO mice compared with WT mice (Fig. 4*Bb*,*Db*,*E*).

**Figure 4-1.** Statistical table for immunohistochemistry. ***F***, Fluorescence intensity of BEGAIN was significantly increased in IB4- and PKCγ-positive areas. Significant difference was indicated in Figure 4*F*.

Figure	Comparison (WT naive vs. WT SNI)	Mann–Whitney *U*	*n*
Fig. 4*F*, itensity of BEGAIN	In IB4 area	0.0487103	31–39 slices from three mice in each group
	In PKCγ area	0.0487103	

To determine the lamina specificity of (SNI-induced) BEGAIN upregulation inside the spinal lamina IIi, we measured the fluorescence intensity of BEGAIN signals in PKCγ-positive and IB4-positive areas in the lamina IIi. We identified a 1.51 ± 0.03– and 1.49 ± 0.03–fold increase (both *p* < 0.05) in the BEGAIN signals in the respective IB4- and PKCγ-positive areas of the spinal dorsal horn after SNI (Fig. 4*E*,*F*; *n* = 31–39 slices from three mice). These results indicate that BEGAIN upregulation was broadly distributed in lamina IIi. Also, no BEGAIN signals were detected in SNI-operated BEGAIN-KO mice (Fig. 4*E*)

**Figure 5.**
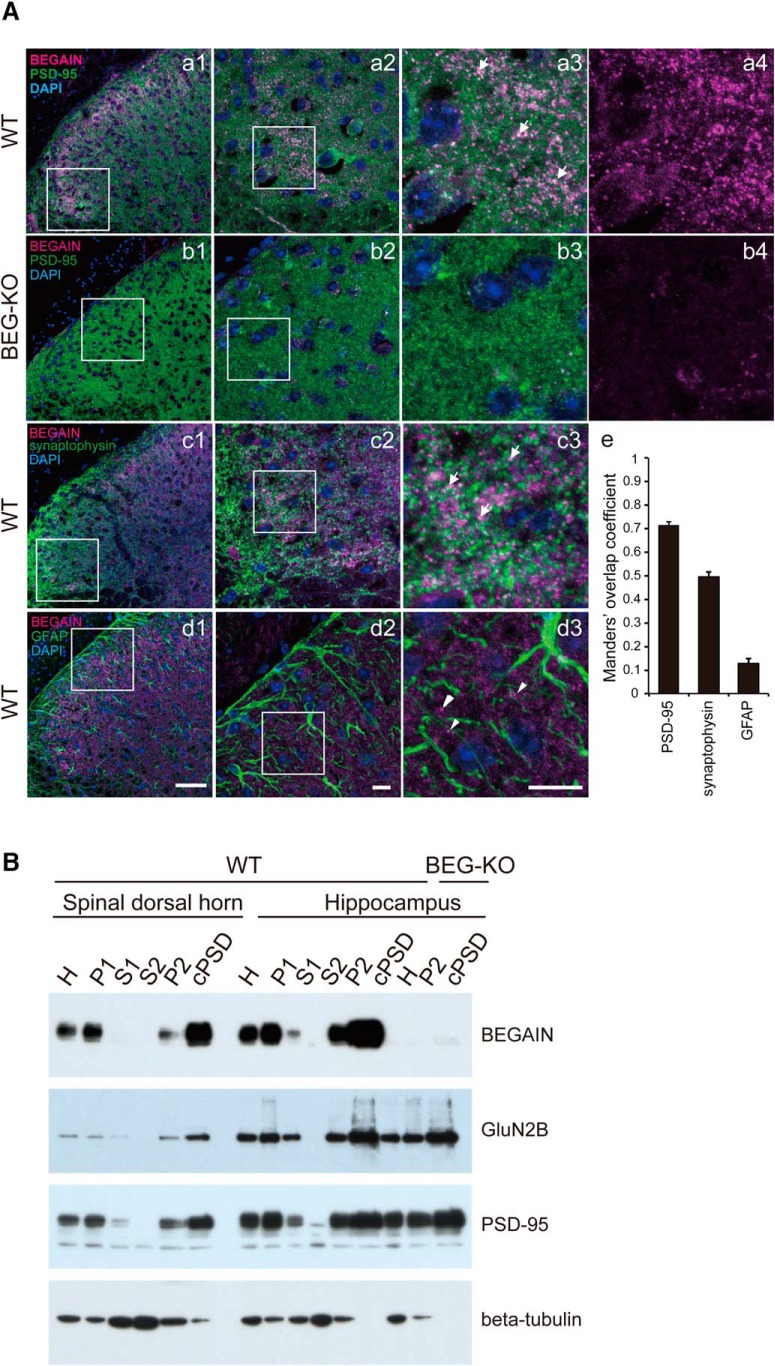
Localization of BEGAIN at the synapses in spinal lamina II. ***A***, Double-staining of BEGAIN and markers. Transverse sections (14 μm) of lumbar spinal cords were prepared from WT (***a***, ***c***, and ***d***) and BEG-KO (***b***) mice. BEGAIN (magenta; ***a–d***), PSD-95, a postsynaptic marker (green; ***a*** and ***b***), synaptophysin, presynaptic marker (green; ***c***) and GFAP, an astrocyte marker (green; ***d***) were detected. Higher magnification of the white boxes in ***a–d1*** are shown in ***a–d2***. Higher magnification views of the white boxes in ***a–d2*** are shown in ***a–d3*** and ***a–b4***. Arrows indicate colocalization signals (***a3*** and ***c3***), and arrowheads indicate BEGAIN signal without GFAP signal (***d3***). Scale bars, 50 μm in ***a–d1*** and 10 μm in ***a–d2*** and ***3***. ***E***, Quantification of colocalization of BEGAIN with markers made by using the Manders overlap coefficient. ***B***, Concentration of BEGAIN in cPSD. Western blot analysis of the subcellular fractions of mouse spinal dorsal horn and hippocampus from WT and BEGAIN-KO (BEG-KO) mice. Spinal dorsal horn (10 μg) and hippocampus (7 μg) proteins were incubated with the anti-BEGAIN, anti-GluN2B, anti–PSD-95, and β-tubulin antibodies. Lane H, homogenate; lane P1, nuclear pellet; lane S1, supernatant 1; lane S2, cytosolic synaptosome; lane P2, crude synaptosomal pellet; cPSD, 0.5% (w/v) Triton X-100–insoluble fraction of P2.

To clarify the localization of BEGAIN at synapses, we immunostained for BEGAIN as well as PSD-95 and synaptophysin, which are markers for post- and presynapses, respectively. BEGAIN was detected in PSD-95–positive (Fig. 5*Aa1*–Aa3, arrows) and synaptophysin-positive (Fig. 5*Ac1–Ac3*, arrows) sites in spinal lamina IIi. On the other hand, it was not detected in GFAP-positive cells, i.e., astrocytes (Fig. 5*Ad1–Ad3*, arrowheads). To confirm the colocalization of BEGAIN and PSD-95 or synaptophysin, we performed Manders overlap coefficient analysis. The fluorescent signals of BEGAIN reliably overlapped with PSD-95 and synaptophysin, but not with GFAP (Fig. 5*Ae*, Manders overlap coefficient 0.71 ± 0.01, 0.50 ± 0.02, and 0.13 ± 0.02, respectively). These results indicate that BEGAIN localized at the synapse in the spinal lamina IIi. To further confirm the distribution of BEGAIN at the synapse of the spinal dorsal horn, we separated six fractions from the spinal dorsal horn biochemically. PSD-95 and GluN2B were highly enriched in the cPSD fraction in both WT and BEGAIN-KO mice (Fig. 5*B*). BEGAIN was detected in the homogenate, P1, P2, and cPSD, but not in soluble fractions (S1 and S2). BEGAIN was highly enriched in the cPSD fraction of the spinal dorsal horn (Fig. 5*B*, upper panel). The subcellular distribution pattern of BEGAIN in the hippocampus was similar to that in the spinal dorsal horn (Fig. 5*B*). These results and those shown in Figure 4 suggest that BEGAIN is a synaptic protein in neurons of the spinal lamina IIi. Furthermore, BEGAIN might be a postsynaptic protein, because BEGAIN was abundant in the PSD fraction and the Manders overlap coefficient for PSD-95 was higher than that for synaptophysin. On the other hand, BEGAIN was not detected in P2 and cPSD in BEGAIN-KO mice, whereas PSD-95 and GluN2B were (Fig. 5*B*).

### EPSC kinetics in lamina II of spinal dorsal horn in WT and BEGAIN-KO mice after SNI

**Figure 6.**
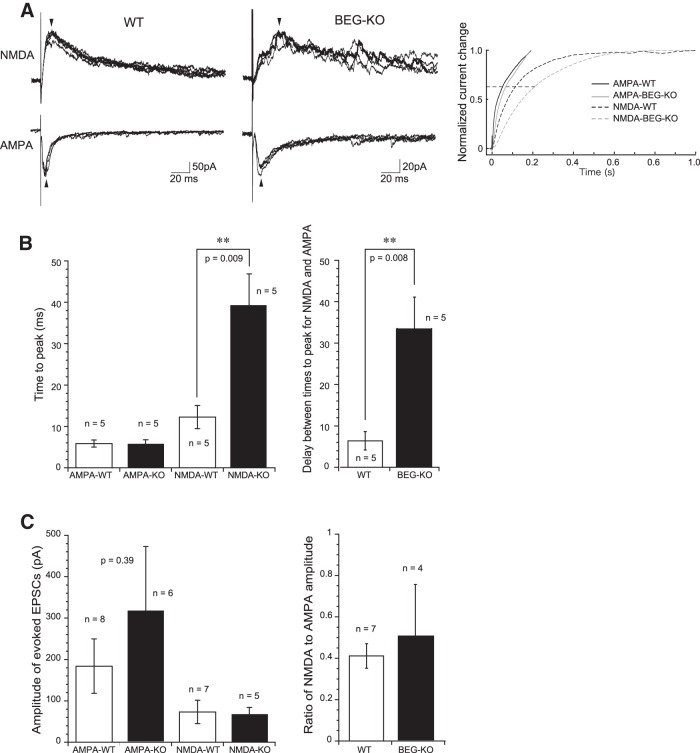
Change in the rising phase of evoked NMDAR EPSCs, but not that of AMPAR EPSCs, in SG neurons of BEGAIN-KO mice. ***A***, Examples of evoked AMPAR and NMDAR EPSCs in WT (left traces) and BEGAIN-KO (BEG-KO; middle traces) mice. NMDAR EPSCs in KO mice had a slower kinetics than those in WT mice. The EPSC amplitude of the traces was not similar. However, there is no significant difference of the EPSC amplitude between the two groups (see Results). Arrows show peaks for the evoked EPSCs. The right graph shows normalized summation of current changes of the traces shown in the left and middle traces. Note that delay between the time constants (0.63, indicated by dashed line) for AMPAR and NMDAR EPSCs in BEG-KO mice is nearly twice that in WT mice. ***B***, Summary showing time to peak for AMPAR and NMDAR EPSCs (left graph), and delay between times to peak for AMPAR and NMDAR EPSCs elicited in single SG neurons (right graph) in WT and BEG-KO mice. ***C***, Amplitude of evoked EPSCs (left graph) and ratio of NMDAR to AMPAR EPSC amplitude (right graph).

To clarify the role of BEGAIN in the channel activity of spinal lamina II neurons after SNI, we analyzed spontaneous EPSCs and evoked EPSCs mediated by α-amino-3-hydroxy-5-methyl-4-isoxazolepropionic acid receptor (AMPAR) and NMDAR in the SG neurons of lamina II (Fig. 6). Whole-cell recordings were obtained from 18 and 17 SG neurons in SNI-operated WT and BEGAIN-KO mice, respectively. There was no difference in input membrane resistance (WT, 362 ± 66 MΩ, *n* = 9; KO, 405 ± 71 MΩ, *n* = 11; *p* > 0.05) or membrane capacitance (WT, 61.1 ± 5.6 pF, *n* = 9; KO, 63.8 ± 8.0 pF, *n* = 11; *p* > 0.05) between WT and BEGAIN-KO mice. Under the voltage-clamp condition at a holding potential of –70 mV, SG neurons examined in both mice exhibited spontaneous EPSCs. The amplitude and frequency of spontaneous EPSCs did not differ between the two groups of mice [frequency, 11.0 ± 2.2 Hz (*n* = 10) in WT, 11.6 ± 2.0 Hz (*n* = 11) in KO, *p* > 0.05; amplitude, 11.4 ± 1.5 pA (*n* = 10) in WT, 11.3 ± 1.2 pA (*n* = 11) in KO, *p* > 0.05]. These results suggest that BEGAIN knockdown did not alter passive membrane properties or spontaneous excitatory synaptic transmission of SG neurons. We then examined evoked excitatory synaptic responses by focal stimulation. The amplitude of evoked EPSCs also did not differ between the two groups (WT, 173 ± 60 pA, *n* = 9; KO, 319 ± 155 pA, *n* = 6; *p* > 0.05) recorded at a holding potential of –70 mV. In the presence of a GABA_A_ receptor antagonist, bicuculline (20 μm); a glycine receptor antagonist, strychnine (2 μm); or a non-NMDA receptor antagonist, CNQX (20 μm), the evoked EPSCs were completely inhibited, indicating that the evoked EPSCs were mediated by AMPA receptors. We further examined evoked NMDAR EPSCs isolated in the presence of these three antagonists at a holding potential of 40 mV, as previously reported (Katano et al., 2008). The NMDAR EPSCs in BEGAIN-KO mice had a shape of currents different from those in WT mice. The former mice showed relatively slower developing currents and did not have the normal rising phase (left and middle traces in Fig. 6*A*). Also, the time to peak of NMDAR EPSCs in BEGAIN-KO mice was significantly longer than in WT mice. However, the time to peak and decay time of AMPA EPSCs (evoked EPSCs recorded at –70 mV) did not differ between WT and BEGAIN-KO mice (left graph in Fig. 6*B*). The delay between times to peak for AMPAR and NMDAR EPSCs elicited in singe SG neurons was also longer in BEGAIN-KO mice than in WT mice (right graph in Fig. 6*B*). The amplitude of NMDAR EPSCs and the ratio of NMDAR to AMPAR EPSCs amplitude did not differ between WT and BEGAIN-KO mice (Fig. 6*C*). These results indicate that slower-developing NMDAR EPSCs in the BEGAIN-KO mice resulted in lengthening of the delay between times to peak for AMPAR and NMDAR EPSCs.

### Attenuation of mechanical allodynia in BEGAIN-KO mice after SNI

**Figure 7.**
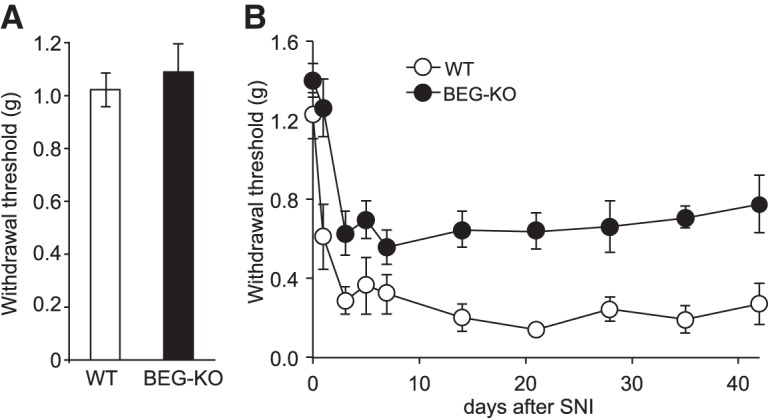
Behavioral analysis of WT and BEGAIN-KO mice after SNI. ***A***, Basal mechanical sensitivity. Withdrawal threshold of naive WT (white column) and BEGAIN-KO (BEG-KO, black column) mice was assessed in 8- to 12-week-old mice. Data are expressed as described in Materials and Methods as the mean ± SEM (*n* = 12–13). ***B***, Time course of paw withdrawal threshold after SNI. Withdrawal thresholds on the ipsilateral side of WT (white circle) and BEG-KO (black circle) mice were assessed before and after SNI. Data are expressed as the mean ± SEM, *n* = 11 (WT) and 14 (BEG-KO). Significant differences were indicated in Source data.

To examine behavioral consequences of altered NMDAR EPSCs in BEGAIN-KO mice, we next assessed mechanical sensitivity of the mice after SNI. In naive mice, there was no significant difference in the withdrawal threshold to mechanical stimuli (WT: 1.02 ± 0.06 g, *n* = 12; BEGAIN-KO: 1.09 ± 0.11 g, *n* = 13) between WT and BEGAIN-KO mice, suggesting that BEGAIN was not involved in nociception in naive mice (Fig. 7*A*). Because thermal allodynia was not observed in the SNI model (Decosterd and Woolf, 2000), we examined the effects of BEGAIN knockdown on mechanical sensitivity. After the SNI operation, the paw withdrawal threshold of both WT and BEGAIN-KO mice markedly dropped on day 3, but the threshold in the BEGAIN-KO mice was significantly higher than that in the WT mice (Fig. 7*B*, WT: 0.29 ± 0.07 g, *n* = 11; BEGAIN-KO: 0.63 ± 0.11 g, *n* = 14). The decreased threshold in each group was maintained after day 7 (WT: 0.32 ± 0.10 g, *n* = 11; BEGAIN-KO: 0.56 ± 0.08 g, *n* = 14), and was retained over the 40-d observation period after SNI (WT: 0.27 ± 0.11 g, *n* = 11; BEGAIN-KO: 0.78 ± 0.15 g, *n* = 14). The decrease in the mechanical threshold was significantly attenuated in BEGAIN-KO mice compared with WT mice after SNI. Collectively, these results suggest that BEGAIN was involved in neuropathic pain after peripheral nerve injury.

**Figure 7-1.** Statistical table. ***B***, Withdrawal threshold at all days after SNI showed a significant difference between WT and BEG-KO. The withdrawal threshold for WT was significantly decreased at 1–42 d after SNI compared with that for WT naive. The withdrawal threshold for BEG-KO was significantly decreased at 3–42 d after SNI compared with that for BEG-KO naive.

	Comparison	Comparison	
Fig. 7*B*, days after SNI	WT vs. BEG-KO, Mann–Whitney *U*	Naive vs. after SNI, Bonferroni	
		WT, *n* = 11	BEG-KO, *n* = 14
0	0.1303		
1	0.0056	<0.01	
3	0.0124	<0.01	<0.01
5	0.0202	<0.01	<0.01
7	0.0193	<0.01	<0.01
14	0.0009	<0.01	<0.01
21	0.0003	<0.01	<0.01
28	0.0044	<0.01	<0.01
35	0.0019	<0.01	<0.01
42	0.0053	<0.01	<0.01
ANOVA: WT	1.36 × 10^–18^		
ANOVA: BEG-KO	2.19 × 10^–9^		

## Discussion

### Comparative analysis of PSD fraction of spinal dorsal horn by iTRAQ-based proteomic approach using Y1472F-KI mice before and after SNI

To screen for neuropathic pain–related proteins in the signaling pathway after the phosphorylation of GluN2B at Y1472, we performed a comparative proteomic analysis using the PSD fraction. From this analysis, we identified 271 PSD proteins, including glutamate receptors, associated signaling proteins, and scaffold proteins. Thirteen proteins were specifically increased in expression in the WT SNI among the four groups (Fig. 1*C*, Table 1, and PASS00929 in Peptide Atlas), indicating that their accumulation to the PSD after SNI depends on the phosphorylation of GluN2B at Y1472. Because calcium influx is attenuated in Y1472F-KI mice (Nakazawa et al., 2006; Matsumura et al., 2010; Katano et al., 2011), it is possible that the increase in expression of these 13 proteins, such as CaMKIIβ, Disk large-associated protein 3 (SAPAP 3), and BEGAIN, depended on the intracellular calcium signaling after SNI. The binding of CaMKII to GluN2B in the synapse is facilitated by autophosphorylated CaMKII (Bayer and Schulman, 2001; Bayer et al., 2006), which requires calcium influx from GluN2B-containing NMDAR (Abe et al., 2005; Nakazawa et al., 2006; Thalhammer et al., 2006; Matsumura et al., 2010; Halt et al., 2012). Activated CaMKIIβ, with activation due to calcium influx, phosphorylates SAPAP, thereby inducing its accumulation at the synapse (Shin et al., 2012). Also, SAPAP is necessary for the recruitment of BEGAIN to PSDs via binding to PSD-95 (Deguchi et al., 1998).

In our proteomic analysis, amounts of Protein piccolo and SynGAP were increased in both WT and Y1472F-KI mice after SNI (Table 1), suggesting that accumulation of these proteins in the PSD during neuropathic pain did not require the phosphorylation of GluN2B at Y1472. Our proteomic analysis using Y1472F-KI mice is an effective design for identification of chronic pain–related proteins in either phosphorylation-dependent or -independent pathways.

### Identification of BEGAIN as a neuropathic pain–related protein in the spinal dorsal horn

Among candidate proteins in our proteomic analysis, we focused on BEGAIN because it specifically increased in WT SNI mice (Table 1 and Fig. 1*D*,*E*) and, furthermore, because its role had not been clarified. BEGAIN was originally identified as a binding protein of PSD-95, and it was shown to be specifically expressed in brain and to be abundant in the PSD fraction (Deguchi et al., 1998; Lim et al., 2003). Our proteomic study supports the possibility that after phosphorylation of GluN2B at Y1472, BEGAIN is transferred to the NMDAR complex of the spinal dorsal horn after peripheral nerve injury, because protein upregulation of BEGAIN in the PSD after SNI was affected by the phosphorylation of GluN2B at Y1472. On the other hand, BEGAIN is transferred to the Triton X-100–insoluble fraction, which is similar to the cPSD fraction, via binding of PSD-95 in CHO cells, and its localization at the synapse is decreased by inhibition of NMDAR in cultured hippocampus neurons (Deguchi et al., 1998; Yao et al., 2002). It is assumed that localization of BEGAIN, as a part of the NMDAR complex, is influenced by activation of NMDARs (Lim et al., 2003). In our present study, mechanical allodynia after SNI was significantly attenuated in the BEGAIN-KO mice. Therefore, we consider that BEGAIN was required for the activities of NMDARs after peripheral nerve injury with the phosphorylation of GluN2B in the spinal dorsal horn. Moreover, BEGAIN and GluN2B were highly enriched in the cPSD not only in the spinal dorsal horn, but also in the hippocampus (Fig. 3*B*), raising the possibility that BEGAIN is also involved in learning and memory after the phosphorylation of GluN2B at Y1472 in the hippocampus.

### Modulation of EPSCs of NMDAR by BEGAIN in spinal lamina II after peripheral nerve injury

Precise control of NMDAR EPSC kinetics is crucial for the maintenance of neural plasticity during neuropathic pain as well as during learning and memory formation (Paoletti et al., 2013). Alterations of NMDAR EPSCs in lamina II neurons, i.e., a slower decay phase of currents, were earlier demonstrated during hyperalgesia and allodynia after sciatic nerve ligation (Iwata et al., 2007). Interestingly, BEGAIN knockdown exhibited slower-developing NMDAR but not AMPAR EPSCs in spinal lamina II, which prolonged the time to peak between NMDAR and AMPAR EPSCs (Fig. 6*A*,*B*). NMDAR is well known to have a voltage-dependent Mg^2+^ block, and therefore its activation is needed for simultaneous activation of AMPAR. The relatively longer delay between times to peak for AMPAR and NMDAR in BEGAIN-KO mice may have caused a reduction in the number of activated NMDARs during glutamatergic synaptic events in SG neurons. It is possible that the attenuated allodynia in the BEGAIN-KO mice was caused by the difference in kinetics of NMDAR EPSCs. It is generally assumed that ligand-binding properties and gating are determined by GluN2 subunit composition (Paoletti et al., 2013; Glasgow et al., 2014). Iwata et al. (2007) demonstrated that the expression of GluN2B in neurons in spinal lamina II is increased by sciatic nerve ligation. However, our proteomic analysis showed that the amount of GluN2A, B, and D subunits in the PSD was not increased after SNI (PASS00929 in Peptide Atlas), suggesting that the altered NMDAR EPSC kinetics in the BEGAIN-KO mice was not due to GluN2 subunit composition. On the other hand, NMDAR current showed high fluctuation in BEGAIN-KO mice. BEGAIN is localized at the synapse and directly binds to PSD-95 (Deguchi et al., 1998). Thus, NMDAR current might show high fluctuation by destabilization of the PSD complex containing NMDAR, higher heterogeneity of the involved channel populations, or a reduction in open probability as a consequence of BEGAIN deletion.

### Involvement of BEGAIN in pathological pain in spinal lamina IIi

The current study revealed that BEGAIN was specifically involved in the transmission of abnormal pain, such as allodynia, but not of physiological pain (Fig. 7). Interestingly, BEGAIN was detected at synapses of PKCγ-positive as well as IB4-positive spinal lamina IIi areas (Fig. 4*B*,*C*). Generally, PKCγ-positive interneurons receive innocuous stimuli via myelinated fibers in the ventral region of lamina IIi and lamina IIIo (Polgár et al., 1999; Braz et al., 2014); however, during allodynia, the activation of sensory fibers, which normally detect touch, elicits abnormal pain via projection neurons of lamina I through the somatostatin- or PKCγ-positive interneurons in laminae IIi–IV (Torsney and MacDermott, 2006; Neumann et al., 2008; Woolf, 2011; Duan et al., 2014; Peirs et al., 2015). Thus, allodynia is maintained by not only laminae I–IIo but also lamina IIi–IV in the spinal dorsal horn circuits (Neumann et al., 2008; Braz et al., 2014; Duan et al., 2014). Our results and previous studies suggest that BEGAIN serves the abnormal pain transmission in lamina IIi via low-threshold myelinated fibers.

Our findings suggest that NMDAR and BEGAIN mutually interacted for the maintenance of pathological pain in spinal lamina IIi because BEGAIN expression in the PSD was increased by NMDAR activity accompanying the phosphorylation of GluN2B at Y1472 after peripheral nerve injury (Table 1), and also because BEGAIN modulated the EPSCs of NMDAR (Fig. 6). Accordingly, the pathological pain circuit in the spinal dorsal horn may be established by the regulatory relationship between NMDAR and BEGAIN. Moreover, BEGAIN knockdown might have led to destabilization of the PSD complex containing NMDAR, suggesting that BEGAIN, through interaction with PSD-95, stabilized the PSD complex. In addition, the N-terminal sequence of BEGAIN includes a part of the F-BAR (FES-CIP4 Homology and Bin/Amphiphysin/Rvs) homology region, which plays critical roles in membrane reorganization via direct binding to cellular membranes (Frost et al., 2009). After the accumulation of BEGAIN at synapses during the neuropathic pain after NMDAR activation, it would seem that BEGAIN may play a role in neural plasticity via its binding to cellular membranes or formation of the PSD complex. Clarification of the molecular function or modifications of BEGAIN in detail may provide a better understanding of the mechanism for pathological pain in the spinal dorsal horn.

## Synthesis

The decision was a result of the Reviewing Editor Michael Fainzilber and the peer reviewers coming together and discussing their recommendations until a consensus was reached. A fact-based synthesis statement explaining their decision and outlining what is needed to prepare a revision is listed below. The following reviewers agreed to reveal their identity: Izhak Michaelevski, Dianna Willis

In this manuscript, the authors perform proteomic analyses of postsynaptic density (PSD) within the spinal dorsal horn to determine downstream mediators that cause neuropathic pain as a result of injury-induced GluN2B phosphorylation. Using a knockin mouse line that demonstrates attenuated neuropathic pain, where the critical residue of GluN2B has been altered (Y1472F; Y1472F-KI), they identify a population of PSD proteins that are only increased in wildtype mice following sciatic nerve injury and not in Y1472F-KI. They concentrate their subsequent studies on one of these proteins, brain-enriched guanylate kinase-associated protein (BEGAIN), whose function in the dorsal horn is unknown. They generate BEGAIN knockout mice and demonstrate attenuated neuropathic pain following sciatic nerve injury, while having no effect on normal mechanical stimuli threshold. Their results suggest a putative role for BEGAIN in neuropathic pain via NMDA receptor activation following GluN2B phosphorylation in the dorsal horn. Overall, this is an interesting paper identifying a potential novel player in pathological pain following peripheral nerve injury. As such, it might be of high interest to many readers. However there are several questions and issues that must be addressed for the manuscript to be suitable for publication, as follows:

Major Issues

1. The rationale for why the authors chose to focus on BEGAIN needs to be better elucidated. Why did they choose this over the other 20 proteins increased following injury in the wildtype compared to the Y1472F-KI? How does the PSD proteome change following injury in the BEGAIN knockout animals. Does knockout of BEGAIN lead to a global change to the proteins that are recruited to the PSD following injury?

2. In the experiments in Figure 1A, the authors should perform intrathecal injection of Ro25-6981 in the Y1472F-KI animals to show specificity of target.

3. Proteomics data

a. There is no assessment of potential analytical bias in the iTRAQ data. Since it is difficult to define biological replicates in such a sample, multiple analytical replicates should be provided for evaluation of the quality of protein quantification.

b. No spike-in experiment was conducted to compensate for global and fold change biases in iTRAQ labeling. The robustness of the evaluation of media average protein ratio is not clear in the absence of median absolute deviation analysis, which was not presented.

c. Why was 20% change defined as the significance threshold for protein expression?

d. No false discovery rate assessment was conducted

e. Several detection parameters were left out: miscleavages, database version and type, modifications types included, mass accuracy, acceptance score cutoff threshold. No info is provided on how database entry redundancies or protein isoforms data were handled?

f. Why did the authors choose MALDI over ESI, which should be more straightforward for this set of the experiment?

g. There is no contamination evaluation for synaptosomes enrichment.

4. Some of the staining is not convincing, and the manuscript could benefit from more rigorous evaluation of antibody specificities and co-localization analyses.

a. The authors do not show specificity tests of their BEGAIN antibodies using Western blots. This should be done and shown.

b. Figure 1D could benefit from both higher resolution and higher magnification images (it is difficult to appreciate the increase in BEGAIN signal from these images). c. In Figure 4, it would be good to see uninjured and post-SNI images of the IB4 and PKCγ layers to determine if there is a global increase in BEGAIN or if the increase is concentrated in a specific layer. Again, higher magnification images would be helpful. d. The colocalization indicated by the staining in Figure 5 isn't convincing. Also, is this a single optical section of a z-stack? A z-stack with colocalization analysis using Pearson's correlation coefficient (or a similar measure) should be performed.

e. Based on distribution of BEGAIN in synaptosomal fractions, the authors conclude that it is synaptic protein in lamina IIi and hippocampus. Colocalization with synaptic proteins in confocal immunohistochemical images would provide clear validation for this claim.

5. The role of the electrophysiological experiment is not clear. It shows time to peak kinetic difference of evoked NMDAR EPSCs between wt and KO mice

a. Although the authors could not find NMDA to AMPA amplitude ratio change, overall, the representative graphs in wt and KO mice are not comparable by the amplitude.

b. At +40 mV, NMDAR current shows high fluctuation, which may be ascribed either to higher heterogeneity of the involved channel populations or due to reduction of open probability or the channel number, current fluctuation is enhanced.

c. The authors did not demonstrate the contribution of NR2B in the current change. Experiments with NR2B inhibitor or current rectification analysis could resolve this issue.

Additional issues regarding clarity of data presentation:

1. The use of bold fonts is disturbing to the eye and makes reading difficult.

2. Figure 1 is overloaded with text.

3. Figure 5A(f) is mislabeled IB4 instead of GFAP.

4. Are the images in figure 5B from a single gel or multiple gels? Methods are not clear.

5. There is no need for colored representation in Fig. 6

6. Axes titles are not similarly formatted and font sizes vary between the graphs, especially on Fig. 6 B&amp;C

7. Fig. 7B - there is no need to show significance symbol over each point. Significance is quite clear visually. Provided the authors want to highlight significance, they should conduct trend analysis for the same curve and statistical analysis of the trend describing fit equation parameters instead comparing each point.

8. Table 1 is a mess. I would recommend to the authors to turn it to supplementary figures

9. Table 2 lacks data:

i. It should include peptide data (sequences, charge states, quantitative data)

ii. The present view of the table does not provide

1. Quantitative data per proteins: median and/or mean, SD or MAD

2. Qualitative data - peptides presented are not clarified whether the number is for unique peptides or detected peptides, what is sequence overlap between peptides

3. Protein detection with a single or even two peptides while there is no analytical and biological replicate cannot be considered as a correct approach

10. The manuscript would benefit from careful editing for English grammar, and to improve clarity of the writing.
